# Drug-drug interactions in subjects enrolled in SWOG trials of oral chemotherapy

**DOI:** 10.1186/s12885-021-08050-w

**Published:** 2021-03-26

**Authors:** Lauren A. Marcath, Colin M. Finley, Siu Fun Wong, Daniel L. Hertz

**Affiliations:** 1 Department of Pharmacotherapy, College of Pharmacy and Pharmaceutical Sciences, Washington State University, Spokane, WA 99203 USA; 2 Department of Clinical Pharmacy, College of Pharmacy, University of Michigan, 428 Church St., Room 3054 College of Pharmacy, Ann Arbor, MI 48109-1065 USA; 3 Chapman University School of Pharmacy, Irvine, CA 92618 USA

**Keywords:** Oncology clinical trial drug interaction

## Abstract

**Background:**

Patients with cancer are at increased risk of drug-drug interactions (DDI), which can increase treatment toxicity or decrease efficacy. It is especially important to thoroughly screen DDI in oncology clinical trial subjects to ensure trial subject safety and data accuracy. This study determined the prevalence of potential DDI involving oral anti-cancer trial agents in subjects enrolled in two SWOG clinical trials.

**Methods:**

Completed SWOG clinical trials of commercially available agents with possible DDI that had complete concomitant medication information available at enrollment were included. Screening for DDI was conducted through three methods: protocol-guided screening, Lexicomp® screening, and pharmacist determination of clinical relevance. Descriptive statistics were calculated.

**Results:**

SWOG trials S0711 (dasatinib, *n* = 83) and S0528 (everolimus/lapatinib, *n* = 84) were included. Subjects received an average of 6.6 medications (standard deviation = 4.9, range 0–29) at enrollment. Based on the clinical trial protocols, at enrollment 18.6% (31/167) of subjects had a DDI and 12.0% (20/167) had a DDI that violated a protocol exclusion criterion. According to Lexicomp®, 28.7% of subjects (48/167) had a DDI classified as moderate or worse, whereas pharmacist review indicated that 7.2% of subjects (12/167) had a clinically relevant interaction. The majority of clinically relevant DDI identified were due to the coadministration of acid suppression therapies with dasatinib (83.3%, 10/12).

**Conclusions:**

The high DDI prevalence in subjects enrolled on SWOG clinical trials, including a high prevalence that violate trial exclusion criteria, support the need for improved processes for DDI screening to ensure trial subject safety and trial data accuracy.

**Supplementary Information:**

The online version contains supplementary material available at 10.1186/s12885-021-08050-w.

## Background

Drug-drug interactions (DDI) can cause treatment to be unsafe for patients by increasing drug toxicity or decreasing treatment efficacy [1]. Patients with cancer have particularly high risk of DDI due to their increasing age, numerous comorbidities and high rates of polypharmacy [2]. An estimated 16–41% of patients receiving cancer treatment have a potential DDI [3–7], which increase risk of severe toxicity nearly three-fold [8]. DDI can be detected using high performing DDI screening tools [9] and effectively managed by incorporating clinical pharmacists or pharmacologists on the healthcare team [4, 5].

DDI can affect drug levels by altering drug absorption, distribution, metabolism or excretion or can affect drug response through mechanistic synergy or antagonism [1]. Cancer treatment is shifting from primarily infusion-based treatment towards oral agents [10]. In addition to the typical concerns with metabolic DDI, oral agents have additional DDI concerns relating to their need to be absorbed from the gastrointestinal tract. Intestinal absorption of oral agents can be affected by changes in gastrointestinal pH and activity of uptake transports. Concomitant administration of gastric acid suppression such as proton pump inhibitors (PPI) or histamine H_2_ antagonists (H2RA) with tyrosine kinase inhibitors can reduce drug absorption decreasing AUC as much as 60% [11], which decreases systemic exposure and treatment efficacy [12, 13]. Additionally, these oral agents are often given daily over an extended period of time increasing the risk of DDI.

DDI management is particularly critical for subjects enrolled in oncology clinical trials, within which the benefits and harms of trial agents are determined. Current processes to detect DDI during trial eligibility screening are inadequate and lack standardization across sites, even within the National Cancer Institute’s National Clinical Trials Network (NCTN) system [14]. Few studies have examined the prevalence of DDI in oncology clinical trial subjects [15, 16]. In our prior work, nearly 25% of subjects enrolled on an NCTN clinical trial at the University of Michigan Rogel Cancer Center were found to have at least one major or contraindicated DDI [16]. This high prevalence suggests many DDI are not being detected and managed during eligibility assessment screening, which raises concerns about trial subject safety and data accuracy.

Based on the high prevalence of DDI in subjects at their time of enrollment on NCTN trials at a single institution, the objective of this study was to determine the prevalence of DDIs involving trial agents in subjects at enrollment in multi-center SWOG clinical trials. A secondary objective was to determine the prevalence of DDI caused by the addition of medications in subjects while on SWOG clinical trials.

## Methods

### Data collection/selection

All closed SWOG clinical trials with available data were evaluated for inclusion. SWOG clinical trials of commercially available agents that collected comprehensive concomitant medication information at the time of enrollment were eligible for inclusion. Trials were excluded if the trial agent did not have any possible DDI. Complete medication lists at enrollment and medication changes during the trial for each subject were collected from the existing trial record. Concomitant medications that were noted to be administered for two or fewer doses were not included in the total number of medications a subject was taking or evaluated during DDI screening.

### Protocol-guided screening

Detailed methods for protocol guided screening have been previously described [16]. Briefly, clinical trial protocols were reviewed for all language discussing concomitant medications with DDI concerns that should be considered exclusion criteria, medications to avoid, or medications to use with caution. Medication lists were compared to this protocol information to determine whether each subject had a DDI according to protocol-guided screening for the trial on which they were enrolled.

### Lexicomp® guided screening

Medication lists were screened for major or contraindicated DDI involving the trial agent using Lexicomp® Drug Interactions. Lexicomp® was selected based on its strong performance when screening for DDI with oral chemotherapy [9].

### DDI clinical relevance determination

DDI identified by protocol or Lexicomp® guided screening were manually reviewed by a pharmacist and student pharmacists for clinical relevance. Clinical relevance was defined as a DDI that would warrant a drug change or discontinuation to ensure subject safety and drug efficacy. This process is similar to the process we used in previous studies to allow for cross-study comparison [16].

### Statistical analysis

The prevalence of DDI by protocol-guided screening, Lexicomp® guided screening, and clinically relevant DDI were calculated for each SWOG trial and combined across trials. The mean, median, and range of medications per subject was also calculated. Statistical analysis was performed using R software.

The primary analysis did not count any DDI involving antacids, as these can be avoided by properly separating timing of administration. A secondary analysis that includes antacids as DDI was also conducted since administration timing information was not available, therefore, these potential DDI cannot be excluded. The following were considered antacids: aluminum hydroxide, magnesium hydroxide, magnesium carbonate, and calcium carbonate dosed as needed.

## Results

### Protocol characteristics and subjects

Two SWOG trials of commercially available agents that had potential DDI and concomitant medication lists were identified. SWOG 0711 (S0711) and 0528 (S0528) were pharmacokinetic trials of dasatinib and everolimus/lapatinib, respectively. S0711 started October 2008 and closed June 2014, and S0528 started September 2006 and closed August 2009. Medications lists were collected when each trial was conducted as a step within protocol procedures and were available for retrospective review for all subjects enrolled on S0711 (*n* = 83) and S0528 (*n* = 84). At enrollment subjects were receiving 0–29 concomitant medications (mean: 6.6, standard deviation: 4.9). Medication additions during the trial occurred in 40.7% (68/167) of subjects, with a mean of 1.9 (standard deviation = 4.8, range 0–23) medications added per subject.

### DDI detected by protocol-guided screening

**Fig. 1:**
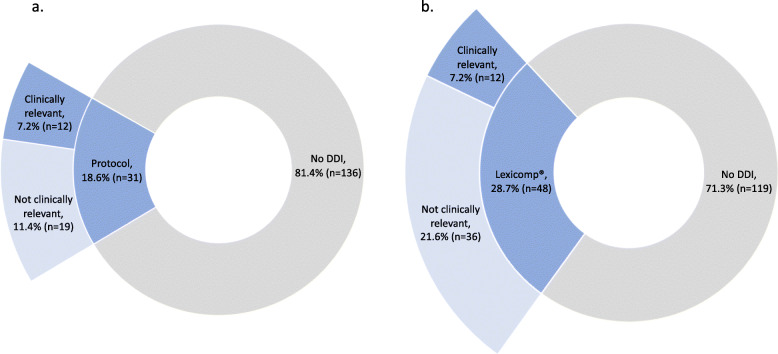
Prevalence of Clinically Relevant Interactions from Lexicomp® and Protocol-Guided Screening. **a**. Protocol-guided screening detected drug-drug interactions (DDI) in 18.6% of subjects. **b**. Lexicomp® detected DDI in 28.7% of subjects. The same subset of interactions detected by protocol-guided screening and Lexicomp® were considered clinically relevant

Protocol-specified concomitant medications that would warrant subject exclusion, or medications that should be avoided or used with caution are shown in Table 1. At the time of enrollment to either of the two trials, 18.6% (31/167, Fig. 1a) of subjects had at least one DDI based on protocol-guidance, the majority of which violated exclusion criteria (12.0% of subjects, 20/167). In the secondary analysis including DDI with antacids, 24.6% (41/167) of subjects had at least one DDI and 17.4% (29/167) of subjects had a DDI that violated exclusion criteria. During the trial, 9.6% of subjects (16/167) had a medication added that was considered a DDI based on protocol guidance. A total of 8.4% (14/167) of subjects had a medication added that violated exclusion criteria.

**Table 1:** Medication or Medication Classes Identified by Protocol Guidance as Drug-drug Interactions

Trial	Exclusion	Avoid	Caution
S0711	Acid suppression therapy (PPI, H2RA, antacid^a^)	CYP3A4 inhibitors, CYP3A4 inducers, antiplatelet agents, anticoagulants	QT prolonging agents
S0528	Acid suppression therapy (PPI, H2RA, antacid^a^)	CYP3A4 inhibitors, CYP3A4 inducers	Warfarin

*PPI* proton pump inhibitor, *H2RA* histamine H_2_ antagonists

^a^antacids were permitted for use if administration separated by 2 h from dasatinib or 1 h from lapatinib per the protocols

In the S0711 trial, 18.1% (15/83) of subjects had at least one DDI at enrollment based on the trial protocol, and 12.0% (10/83) of subjects had a DDI that was a violation of an exclusion criterion (Table 2). Most of these exclusion criteria violations were due to the combination of dasatinib with a PPI (80%, 8/10, Online Resource 1) and the rest were due to an H2RA (20%, 2/10). Including antacids as DDI, 22.9% (19/83) of subjects had an exclusion criterion violation at enrollment. A medication that violated an exclusion criterion was added during the trial in 13.3% (11/83) of subjects, all of which were PPI.

**Table 2:** Drug-drug Interaction Prevalence

Primary analysis						
	Study ID	Protocol: Exclude	Protocol: Avoid	Protocol: Caution	Lexicomp®	Clinically Relevant
DDI At Study Enrollment	S0711	12.0% (10/83)	6.0% (5/83)	2.4% (2/83)	55.4% (46/83)	12.0% (10/83)
	S0528	11.9% (10/84)	3.6% (3/84)	6.0% (5/84)	2.4% (2/84)	2.4% (2/84)
DDI Added During Study	S0711	13.3% (11/83)	6.0% (5/83)	2.4% (2/83)	20.5% (17/83)	13.3% (11/83)
	S0528	3.6% (3/84)	0.0% (0/84)	0.0% (0/84)	0.0% (0/84)	0.0% (0/84)
Secondary analysis						
	Study ID	Protocol: Exclude	Protocol: Avoid	Protocol: Caution	Lexicomp®	Clinically Relevant
DDI At Study Enrollment	S0711	22.9% (19/83)	6.0% (5/83)	2.4% (2/83)	55.4% (46/83)	22.9% (19/83)
	S0528	11.9% (10/84)	3.6% (3/84)	6.0% (5/84)	2.4% (2/84)	2.4% (2/84)
DDI Added During Study	S0711	15.7% (13/83)	6.0% (5/83)	2.4% (2/83)	20.5% (17/83)	15.7% (13/83)
	S0528	6.0% (5/84)	0.0% (0/84)	0.0% (0/84)	0.0% (0/84)	0.0% (0/84)

*DDI* drug-drug interaction

In the S0528 trial, 20.2% (17/84) of subjects had at least one potential DDI at enrollment based on protocol-guided screening (Table 2). The majority of DDI violated protocol exclusion criteria (11.9%, 10/84); these were nearly evenly split between the combination of lapatinib with PPIs (60.0%, 6/10) and H2RAs (50.0%, 5/10). No subjects were taking antacids at baseline, so the results of the secondary analysis were the same as the primary analysis. Three subjects had a protocol identified DDI added while on trial (3.6%, 3/84) and each of these DDI violated an exclusion criterion.

### DDI detected by Lexicomp®

At baseline, 28.7% of subjects (48/167, Fig. 1b) had at least one major or contraindicated DDI detected by Lexicomp®. The majority of these interactions were detected in S0711 and were due to the combination of dasatinib with acid suppression therapies and/or acetaminophen (acid suppression only: 13/46, acetaminophen only: 20/46, both: 11/46, other: 2/46). During the trials 10.2% (17/167) of subjects had a medication added that caused a DDI, all of which were S0711 subjects.

### Clinically relevant DDI

In the primary analysis, 7.2% of subjects (12/167) had at least one DDI at enrollment that was considered to be clinically relevant, and this increased to 12.6% (21/167) when including antacids in the secondary analysis. The majority of these clinically relevant interactions (83.3%, 10/12) were between dasatinib and PPIs or H2RAs. The clinically relevant interactions with lapatinib/everolimus were with verapamil (*n* = 1) and fluconazole (n = 1). The interaction of dasatinib with acid suppression therapy was considered clinically relevant, so 6.6% (11/167) of subjects, all on S0711, had a drug added during the trial that led to a clinically relevant DDI.

## Discussion

Patients with cancer have a high prevalence of DDI [3–6] that can decrease patient safety and increase toxicity [1]. Oncology clinical trial subjects also have high prevalence of DDI due to the lack of standardized screening procedures [14]. In our previous work, approximately 25% of subjects enrolled on an NCTN trial at a single-site had DDI at enrollment [16]. This follow-up analysis of subjects enrolled on SWOG trials across sites confirmed a high prevalence of DDI, though the exact estimate depends on whether the determination is based on the protocol (19–25%), is limited to protocol exclusion criteria (12%), or is based on Lexicomp® (29%) or clinical judgement (7%). This study also found inadequacies in DDI screening for drugs added while a subject is on a clinical trial.

The prevalence of at least one major or contraindicated DDI at enrollment detected by Lexicomp® (29%) is similar to the prevalence detected in subjects enrolling on NCTN trials at UM Rogel Cancer Center (24.2%) [16] and within the ranges previously reported in patients with cancer (16–41%) [3–6]. Direct comparison of these rates should be done cautiously as the prevalence of DDI is largely determined by the interaction potential of the agents used in the trials included in the analysis. This analysis included two trials of agents with numerous DDI, whereas our prior analysis included subjects enrolled in 35 trials with a variety of trial agents and DDI potential. Nevertheless, these findings suggest that the ineffectiveness of DDI screening for oncology clinical trial enrollment is not limited to a single or subset of institutions but is a systemic issue across sites. Based on manual pharmacist review, 7% of subjects had a DDI that was considered clinically relevant, further supporting the conclusion that improved DDI screening is necessary to prevent harm in clinical trial subjects and ensure accuracy of trial data.

The vast majority of DDI detected in these trial subjects were DDI that prevent drug absorption [17]. Absorption DDI are common for oral medications, which are being used more often in cancer treatment due to their improved convenience over parenteral administration [18]. Dasatinib absorption decreases with increasing pH [19], consequently, acid suppression therapy (e.g., PPIs, H2RAs, and antacids) decreases absorption of dasatinib leading to an AUC decrease of between 43 and 61% [11]. Reduced drug absorption leads to lower systemic concentrations that could cause dasatinib treatment efficacy to be decreased, as has been shown for erlotinib and pazopanib [12, 13, 20]. This DDI is particularly concerning given that the primary objective of S0711 was to investigate the pharmacokinetics of dasatinib. This is just one of many possible scenarios where ineffective DDI screening can meaningfully affect the accuracy of the data collected within a clinical trial.

The high DDI prevalence in oncology trial subjects is likely due to the lack of standard DDI screening procedures during trial enrollment eligibility assessment [21]. In our prior survey of SWOG sites, most sites reported that DDI screening relies primarily on DDI guidance within the trial protocol and approximately half of sites indicated that DDI screening is only conducted for DDI that are explicit trial exclusion criterion [14]. Despite sites self-reported reliance on protocols and particular attention to exclusion criteria, in this analysis 12% of subjects had DDI at enrollment that warranted trial exclusion and 7% had a medication added during the trial that warranted trial exclusion. This is perhaps the strongest evidence of the inadequacy of the current systems for DDI screening within oncology clinical trials. One solution that has been proposed is to have pharmacist-led comprehensive DDI screening for all oncology clinical trial subjects [21], however, only 17% surveyed SWOG sites reported pharmacists currently conduct DDI screening. Most sites rely on clinical research coordinators (56%) and study nurses (45%) who may have insufficient knowledge and training on DDI [22, 23], and pharmacist-led DDI screening likely is not given the lack of pharmacists at up to 6% of sites that enroll subjects on SWOG clinical trials [14]. An alternative approach that we have advocated is to deploy a DDI screening tool designed specifically to assist clinical trial staff with screening for DDI for trial subjects [24]. Standardizing DDI screening procedures for clinical trial subjects, either through pharmacist involvement [25] or developing a point of care tool, could dramatically improve the effectiveness and efficiency of DDI screening, yielding significant benefits to trial subjects, staff, and investigators.

A limitation of this study is that only two trials were included. Few SWOG protocols require collection of concomitant medication information, limiting the availability of the data necessary to more comprehensively investigate the prevalence of DDI in SWOG trial subjects. Additionally, the medication lists could not be further verified from what was recorded as part of the original study protocol. The medication lists were likely collected by multiple individuals across trial sites, and it is possible inaccuracies exist. The concomitant medication data did not specify administration times, so it is unknown whether antacid interactions should have been included. Antacids accounted for 50% of the DDI that were classified as exclusion criteria or clinically relevant, consequently, our estimated DDI prevalence is somewhat sensitive to whether it can be assumed that timing of antacid administration was appropriate. Additionally, some subjects had multiple strengths or routes of administration of the same medication. These were treated as individual DDI occurrences since the route and dose of medications can impact the likelihood of a DDI. Finally, we used our standard approach of manual review by a pharmacist to determine DDI clinical relevance; however, slightly different prevalence estimates would likely have been obtained if clinical relevance was determined by a different pharmacist.

## Conclusions

DDI in clinical trial subjects have the potential to adversely affect subject safety and compromise trial data accuracy. Our results confirm a high prevalence of DDI in subjects enrolled on SWOG clinical trials, further supporting the need for improvements to DDI screening procedures, particularly for trials of drugs that have high DDI potential including oral anti-cancer agents.

## Supplementary Information

**Additional file 1.** Drug-drug Interactions Identified at Baseline and Added After Enrollment. Table containing all drug-drug interactions identified at baseline and after enrollment by screening through protocol guidance, Lexicomp®, and by pharmacist review for clinical relevance.

## Data Availability

The datasets generated during the current study are not publicly available but are available upon reasonable request from the corresponding author upon reasonable request and with permission of SWOG.

## Abbreviations

DDI: Drug-drug interaction
NCTN: National Clinical Trial Network
PPI: Proton pump inhibitors
H2RA: Histamine H_2_ antagonists
