# Supplementary material for: Drug-drug interactions in subjects enrolled in SWOG trials of oral chemotherapy
Source: BMC Cancer. 2021 Mar 26;21:324. doi: 10.1186/s12885-021-08050-w (PMC7995697; doi:10.1186/s12885-021-08050-w)
Supplement: Supplementary file 1 — Additional file 1. Drug-drug Interactions Identified at Baseline and Added After Enrollment. Table containing all drug-drug interactions identified at baseline and after enrollment by screening through protocol guidance, Lexicomp®, and by pharmacist review for clinical relevance. [file 12885_2021_8050_MOESM1_ESM.docx]

# **Drug-drug interactions in subjects enrolled in SWOG trials of oral chemotherapy.**

**Journal Name:** Drug Safety

^1^Lauren A Marcath, PharmD

^2^Colin M Finley

^3^Siu Fun Wong, PharmD

^2^Daniel L Hertz, PharmD, PhD (DLHertz@med.umich.edu)

**Affiliations:**

^1^Washington State University, College of Pharmacy and Pharmaceutical Sciences, Department of Pharmacotherapy, Spokane, WA, United States, 99203

^2^University of Michigan, College of Pharmacy, Department of Clinical Pharmacy, Ann Arbor, MI, United States, 48109-1065

^3^Chapman University School of Pharmacy, Irvine, CA, 92618

# **Supplementary Table 1. Drug-drug Interactions Identified at Baseline and Added After Enrollment.**

| Study Agent(s) | Interacting Drug Class | Interaction Drug (n with interaction) | n with Interaction at Enrollment | n with Interaction After Enrollment | Protocol Guidance | Lexicomp® | Clinically relevant |
| --- | --- | --- | --- | --- | --- | --- | --- |
| dasatinib | Proton pump inhibitor | dexlansoprazole | 1 | 0 | Exclude | Yes | Yes |
|  |  | esomeprazole | 1 | 1 | Exclude | Yes | Yes |
|  |  | lansoprazole | 2 | 2 | Exclude | Yes | Yes |
|  |  | omeprazole | 2 | 0 | Exclude | Yes | Yes |
|  |  | pantoprazole | 2 | 8 | Exclude | Yes | Yes |
|  | Histamine H_2_ antagonists | cimetidine | 1 | 0 | Exclude | Yes | Yes |
|  |  | famotidine | 0 | 1 | Exclude | Yes | Yes |
|  |  | ranitidine | 1 | 0 | Exclude | Yes | Yes |
|  | Antacids | aluminum hydroxide | 0 | 1 | Exclude | Yes | Yes |
|  |  | calcium carbonate | 5 | 1 | Exclude | Yes | Yes |
|  |  | milk of magnesia | 5 | 1 | Exclude | Yes | Yes |
|  | Antiplatelet/anticoagulant | clopidogrel | 1 | 0 | Avoid | No | No |
|  |  | enoxaparin | 2 | 2 | Avoid | No | No |
|  |  | warfarin | 2 | 0 | Avoid | No | No |
|  | Corticosteroid | dexamethasone^a^ | 0 | 3 | Avoid | No | No |
|  | Antifungal | fluconazole | 0 | 1 | Avoid | No | No |
|  | Antipsychotic | chlorpromazine | 0 | 3 | Caution | Yes | No |
|  | Analgesic | methadone | 2 | 0 | Caution | Yes | No |
|  |  | acetaminophen | 5 | 1 | None | Yes | No |
|  |  | hydrocodone/acetaminophen | 26 | 5 | None | Yes | No |
|  |  | oxycodone/acetaminophen | 4 | 0 | None | Yes | No |
|  | Dietary supplement | calcium supplement | 7 | 0 | None | Yes | No |
| everolimus/lapatinib | Proton pump inhibitor | lansoprazole | 2 | 0 | Exclude | No | No |
|  |  | omeprazole | 2 | 2 | Exclude | No | No |
|  |  | pantoprazole | 0 | 1 | Exclude | No | No |
|  |  | rabeprazole | 2 | 0 | Exclude | No | No |
|  | Histamine H_2_ antagonists | famotidine | 3 | 0 | Exclude | No | No |
|  |  | ranitidine | 2 | 0 | Exclude | No | No |
|  | Antacid | calcium carbonate | 0 | 1 | Exclude | No | No |
|  |  | milk of magnesia | 0 | 1 | Exclude | No | No |
|  | Antifungal | fluconazole | 1 | 0 | Avoid | Yes | Yes |
|  | Calcium channel blocker | verapamil | 1 | 0 | Avoid | Yes | Yes |
|  | Corticosteroid | dexamethasone^a^ | 1 | 0 | Avoid | No | No |
|  | Anticoagulant | warfarin | 5 | 0 | Caution | No | No |

^a^dexamethasone >1.5mg per day considered inducer of CYP3A4 per protocol
